# Perspective on diffuse light in tissue: subsampling photon populations

**DOI:** 10.1117/1.JBO.26.7.070601

**Published:** 2021-07-02

**Authors:** Samuel S. Streeter, Steven L. Jacques, Brian W. Pogue

**Affiliations:** a Thayer School of Engineering at Dartmouth, Hanover, New Hampshire, United States; b University of Washington, Department of Bioengineering, Seattle, Washington, United States

**Keywords:** diffusion, diffuse light, subdiffuse light, tissue optics

## Abstract

**Significance:** Diffuse light is ubiquitous in biomedical optics and imaging. Understanding the process of migration of an initial photon population entering tissue to a completely randomized, diffusely scattered population provides valuable insight to the interpretation and design of optical measurements.

**Aim:** The goal of this perspective is to present a brief, unifying analytical framework to describe how properties of light transition from an initial state to a distributed state as light diffusion occurs.

**Approach:** First, measurement parameters of light are introduced, and Monte Carlo simulations along with a simple analytical expression are used to explore how these individual parameters might exhibit diffusive behavior. Second, techniques to perform optical measurements are considered, highlighting how various measurement parameters can be leveraged to subsample photon populations.

**Results:** Simulation results reinforce the fact that light undergoes a transition from a non-diffuse population to one that is first subdiffuse and then fully diffuse. Myriad experimental methods exist to isolate subpopulations of photons, which can be broadly categorized as source- and/or detector-encoded techniques, as well as methods of tagging the tissue of interest.

**Conclusions:** Characteristic properties of light progressing to diffusion can be described by some form of Gaussian distribution that grows in space, time, angle, wavelength, polarization, and coherence. In some cases, these features can be approximated by simpler exponential behavior. Experimental methods to subsample features of the photon distribution can be achieved or theoretical methods can be used to better interpret the data with this framework.

## 1 Introduction

The process of light becoming diffuse is the progression from an organized state to a fully disorganized state, through a series of elastic scattering events that spread photon distributions with respect to several physical properties. Each individual scattering event is physically based, with a stochastic probability density function that describes the range of possible outcomes. The transition to diffusion is driven by a multi-event sequence of these individual steps, whereby the full range of initial states becomes increasingly distributed to partially and then fully randomized. The state of the system, e.g., in time and space, can be modeled with basic principles of the underlying physics. When each event is taken individually, the probability of a scatter event is binomial in nature (i.e., scatter versus no scatter), although the physical outcome of the event is distributed across a wide probability of outcome states (i.e., the angle of scattering as dictated by the scattering phase function). The expansion of this process to multiple events provides a Poisson distribution of each state. After many scattering events, the state of the system can be approximated by a Gaussian distribution following the central limit theorem and in many cases, by simple exponential behavior.^1^ Modeling the spread of the state coordinates as ordinate parameters can provide an analytical methodology to interpret the data or design measurements.

**Fig. 1:**
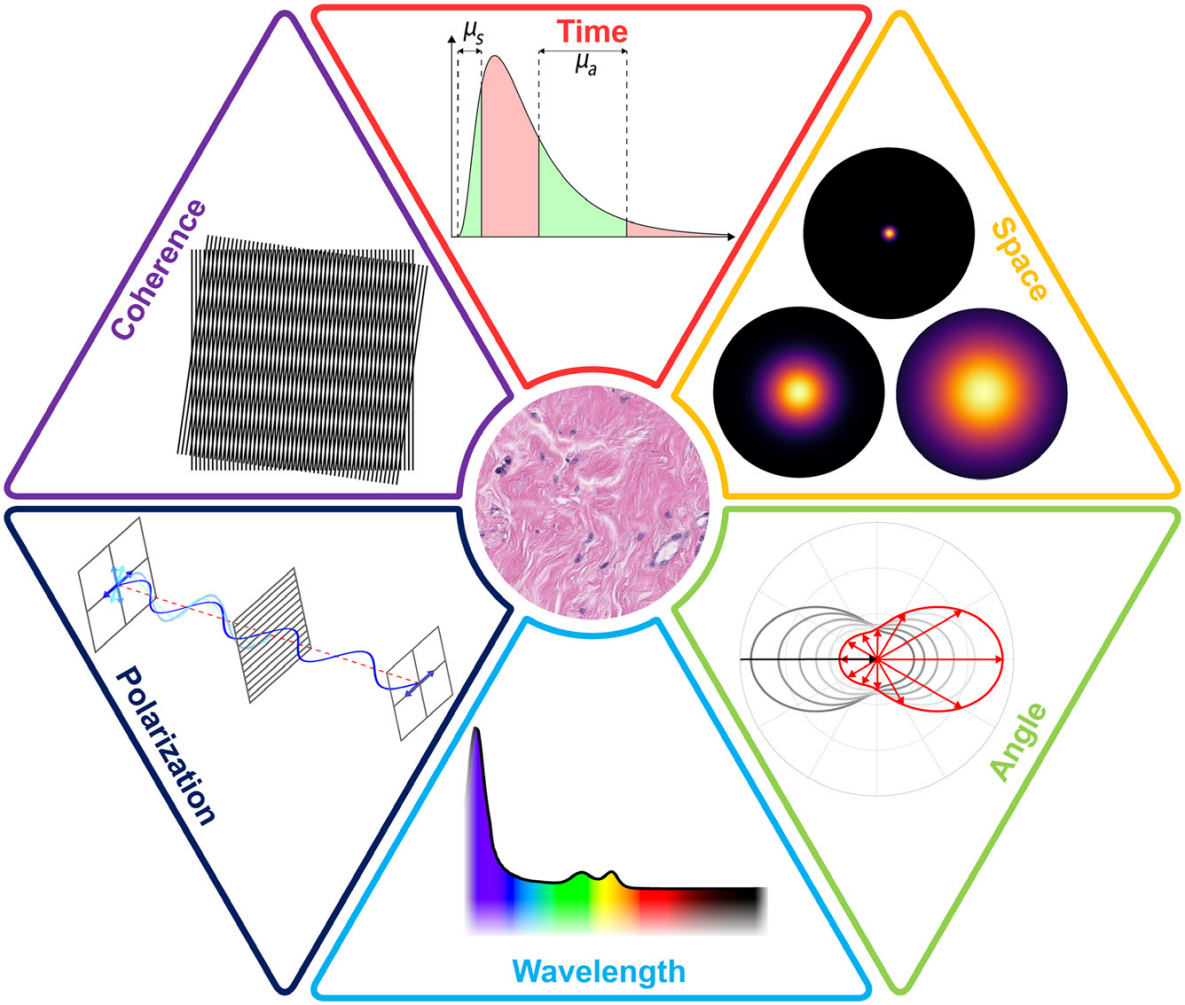
Measurement parameters of light that are affected by the process of diffusion.

Early characterization of light diffusion in tissue came with the empirical observation of a spatial spread over several centimeters that appears diffuse. Analytical modeling of the underlying physics suggested that it could be treated as a random walk, typical of early radiation transport models.^2^ The extension of random walk theory to many events leads to a diffusive appearance both empirically in experiment and analytically in theory.^3^ The extension of this concept of multiple scattering leading to a population distribution that is Poisson or Gaussian in shape can be applied to many measurement parameters of light, including spatial, temporal, and angular distributions, wavelength dispersion, electromagnetic polarization, and phase coherence. These six parameters are illustrated in Fig. 1. In this perspective article, the theory of how one can predict the transition of light from an initially organized state to an increasingly randomized state is explored generally, with potential application to each of these individual parameters.

Modeling of macroscopic light diffusion in tissue is well developed, and the modeling of individual subpopulations of light has been developed in specific applications with insightful analytic modeling.^1^ Individual photon interactions occur on a microscopic scale and include both absorption and scattering events. Scattering specifically must be present for light diffusion to occur and is dependent on index of refraction fluctuations in the tissue that are 10 s of nm to 10 s of μm in scale.^4^ The linkage between these individual microscopic events and the macroscopic distribution of light provides key insight that can be used to measure biophysically relevant features of the tissue. Model-based interpretation of measurements resolved with respect to one or more parameters of light (Fig. 1) can be used to quantify tissue features, such as absorption and scattering coefficients or chromophore concentrations. Model-based interpretations can also inform the design of instrumentation to provide maximal sensitivity to signals with the highest dynamic range to the underlying biophysics of the tissue, such as scatter angle, coherence loss, or polarization change.

**Fig. 2:**
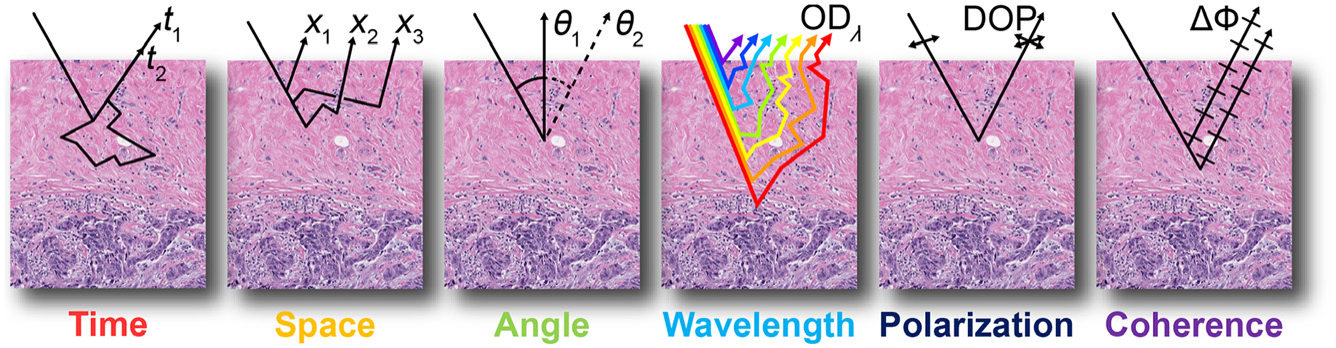
Measuring the six parameters of light, where $t_{i}$ is the time, $x_{i}$ is the spatial position, $\theta_{i}$ the is the angular trajectory, ${\mathrm{OD}}_{\lambda }$ is the wavelength-dependent optical density, DOP stands for degree of polarization, and ΔΦ is the difference in phase between light waves.

Light transport in tissue adheres to three regimes: initial non-scattering or non-diffuse transport, subdiffuse transport, and fully diffuse transport. Although diffuse light in tissue is the bedrock of biomedical optics, challenges inherent to diffuse light transport limit its use in some applications. Specifically, as a turbid medium, tissue diffusely scatters light, resulting in a blurred or low-pass filtered optical signal. This filtering effect transcends different measurement parameters of light (Fig. 1). Although diffuse light contains a wealth of information about the interrogated tissue, this information is limited by reduced resolution in time, space, angular trajectory, etc. Thus, methods of subsampling populations of photons can improve measurement resolution. Figure 2 illustrates the six parameters of light in the context of common measurement schemes to perform photon population subsampling. In Sec. 2, Monte Carlo simulations illustrate the diffusive behavior of light in a scattering medium, and a general analytical expression is given for describing diffusion of an individual parameter of light. Section 3 provides a general framework for categorizing the different ways in which subdiffuse or fully diffuse photon populations can be sampled.

Central to this article is the distinction between the diffusive behavior of light in tissue and distributed parameters of light in tissue. “Diffusion” implies a process of migration in some parameter space (e.g., space, time, or angle). “Diffuse” light might refer to multiply scattered light that has achieved some distribution of parameters, but this is often considered a static state rather than a dynamic, continuous process. Distributed parameters in and of themselves lack the active progression from an organized, well-characterized state to a randomized state. For example, light reflected from a rough metal surface may be characterized by a distribution of photon angular trajectories, and thus, the reflectance is diffuse in appearance. However, the metal is not a diffuse medium. A turbid medium, one that allows the penetration of light and contains a suspension of particles that act as scattering sites (i.e., index of refraction fluctuations), is required for light diffusion to occur. The key distinction is between the process of diffusion of light and the distributed parameters of light; light diffusion is a sufficient but not necessary condition leading to distributed parameters of light.

## 2 Diffusive Transition from an Initial State to a “Diffuse” State

**Fig. 3:**
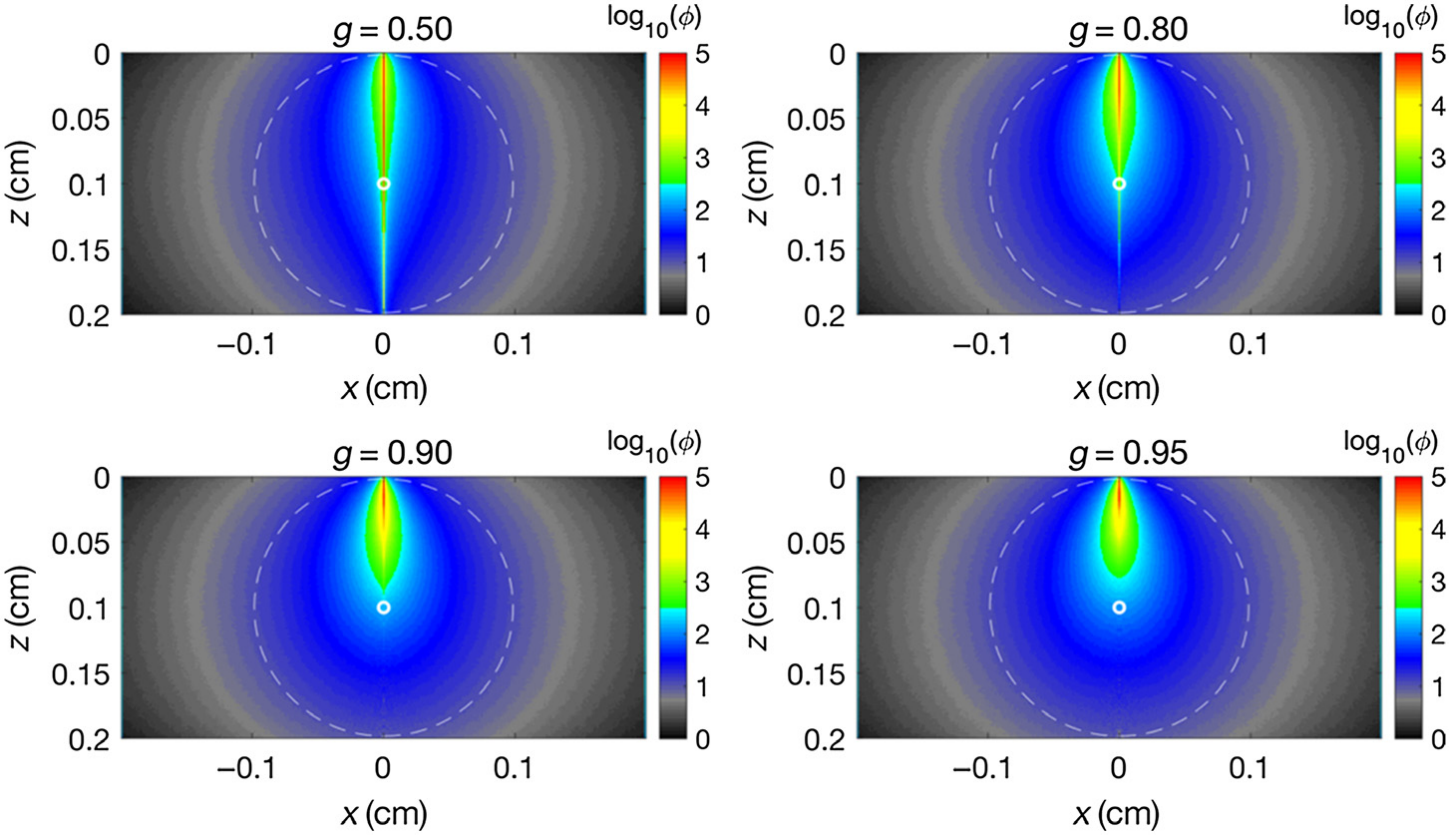
The spread of light within a tissue in response to a very narrow collimated beam, where $\mu_{s}(1-g)$ is held constant while the scattering anisotropy parameter g and scattering coefficient $\mu_{s}$ are varied. g was varied as 0.5, 0.8, 0.9, and 0.95, whereas $\mu_{s}$ varied as 20, 50, 100, and $200\;{\mathrm{cm}}^{-1}$, such that $\mu_{s}(1-g)$ remained a constant $10\;{\mathrm{cm}}^{-1}$. The absorption coefficient was held constant at $\mu_{a}=1\;{\mathrm{cm}}^{-1}$; the tissue was semi-infinite; and the surface boundary was refractive index matched. The dashed line indicates an isoline at a constant fluence (φ) of $10\;{\mathrm{cm}}^{-2}$, beyond which photon diffusion down concentration gradients acts as though the source were at x, z=0.0, 0.1 (i.e., the small, white circle at a depth of one transport mean free path). Colormap fluence field values were scaled logarithmically for improved visualization of the range.

Figure 3 shows the spread of a narrow beam penetrating into a light-scattering tissue, simulated using the MCML codebase.^5^ The primary beam attenuates due to scattering by the tissue. The scattered photons then spread in the tissue yielding a superficial zone of strong fluence. Within this zone, the photons are not “diffuse” but still oriented in response to the initial trajectory of the launched beam; thus, they are “subdiffuse” photons. Distant from this zone, the light attenuates as a function of distance from a central point at $z=1/(\mu_{s}(1-g))$, a depth equal to the transport mean free path, which is 0.1 cm in this example. In this outer zone, the photon trajectories are randomized, and photons are effectively diffusing downward along concentration gradients,^1^ so the photons can be called “diffuse.”

In this figure, the parameters $\mu_{a}$ and $\mu_{s}(1-g)$ are held constant at 1 and $10\;{\mathrm{cm}}^{-1}$, respectively, whereas four pairs of g and $\mu_{s}$ are tested: 0.50, $20\;{\mathrm{cm}}^{-1}$; 0.80, $50\;{\mathrm{cm}}^{-1}$; 0.90, $100\;{\mathrm{cm}}^{-1}$; and 0.95, $200\;{\mathrm{cm}}^{-1}$. Holding $\mu_{a}$ and $\mu_{s}(1-g)$ constant results in the diffuse pattern distant from the source being equivalent in all cases. However, as g increases, $\mu_{s}$ also increases. Therefore, photons undergo more scattering events before reaching the depth $1/(\mu_{s}(1-g))$, allowing multiple scattering to broaden the beam. The photons in this “subdiffuse” region near the source are not yet “diffuse” since they are predominantly still moving forward, down into the tissue rather than diffusing down concentration gradients. However, their trajectories are spreading in trajectory-angle space by a diffusive process.

For low g (see g=0.50), the $\mu_{s}$ is also low, which allows some of the on-axis primary beam to penetrate unscattered into the tissue (red line along z at x=0). However, as g increases, $\mu_{s}$ also increases, and the primary beam is more quickly scattered into a diffusive process. For g=0.8, 0.9, and 0.95, the shapes of the zone of strong fluence become increasingly similar as g increases. Importantly, a variety of parameters may characterize this “subdiffuse” zone such as spatial variation of escaping flux, angles of escaping flux, time course of escaping flux, photon pathlength (and coherence) in escaping of flux, and polarization of escaping flux.

Notably, Monte Carlo simulations are not the only method of illustrating diffuse light transport in tissue. One can simply illuminate human skin and observe diffusely scattered reflectance. Limitations associated with Monte Carlo simulation of light transport are tied to the underlying assumptions in each simulation. In Fig. 3, a key assumption is that of a homogeneous medium, which is rarely if ever true in biological tissue. Nevertheless, the simulations in Fig. 3 demonstrate the impact of tuning optical scattering properties and the transitions between non-diffuse, subdiffuse, and fully diffuse transport, concepts central to this perspective article.

Diffusion can describe the process of an initial amount ($U_{0}$) of a parameter spreading in a parameter space x:

$$C(x,t)=U_{0}\frac{e^{-\frac{x^{2}}{4D_{x}t}}}{\sqrt{4\pi D_{x}t}},$$ (1)

where C(x,t) is the 1D “concentration” of the parameter (amount/unit), i.e., amount at value x per incremental dx at time t, and $D_{x}$ is the diffusivity of the parameter in the x space (${\text{units}}^{2}/s$). (Note that the spatially resolved diffusion may have time-dependence or be steady-state.^1^) The integral

$$\int_{0}^{\infty }C(x,t)dx=1$$ (2)

is true for all times. The generic term “${\text{units}}^{2}/s$” is used, because diffusivity could relate to a spread in some parameter, such as angular trajectory (${\text{radians}}^{2}/s$) or pathlength (${\text{pathlength}}^{2}/s$). Sometimes, a loss process such as absorption is also present, for which an additional term, $\exp(-\mu_{a}ct)$, multiplies Eq. (1), where c is the speed of light in the medium. The key lesson emphasized here is that a diffusive process can describe the transition of a photon from an initial state to a truly “diffuse” state, and this process applies to various measurement parameters of light.

Collected photons will usually involve a distribution of pathlengths through the tissue, which consequently spread the attenuation of collected light. The absorption spectra of a chromophore in the tissue will be distorted by this spread of attenuation, and therefore, reflectance with respect to wavelength is also subject to distortion by a diffusive process.

## 3 Application Techniques of Study in Populations of Photons

The ways in which subpopulations of light can be sampled from tissue can be described in several ways, but a useful way to categorize them is active source-encoding, detector-encoding, and population-encoding (i.e., external tagging) of the signals. Each of these encoding schemes is described in the following subsections.

### 3.1 Source-Encoding Methods

The term “source-encoding” here indicates that the imaging technique leverages a specifically designed source to isolate a desired optical signal. Widespread advances in photonics technology have created a cornucopia of ways in which light can be encoded by a unique source. The use of custom light sources can be chosen to selectively enhance or focus any of the six parameters described in Sec. 1. The most obvious are factors such as wavelength choices, but this can be further augmented by methods such as wavelength-encoding coupled to time^6^ or space^7^ or angle of incidence.^8^ Careful design of methods to match the source-encoding with detector-decoding can allow for intelligent design of diffuse or subdiffuse spectroscopy while avoiding some of the less desirable aspects of diffusion. Polarization-encoding of the source is a technique that persists only through one or two scattering events but can be extremely effective when coupled to sequential subtraction methods to remove diffuse light from an image.^9^ In comparison, coherence source-encoding is a technique widely used in both optical coherence tomography (OCT) and diffuse correlation spectroscopy (DCS), whereby the key to success has been the innovation of using long coherence length light sources that match the length scale of need. In the case of OCT, the coherence length defines the axial spatial resolution in Fourier domain methods over short-path lengths in tissue (d∼1  mm).^10^ However in DCS, the coherence length of the speckle pattern, dictated by underlying hemodynamics, determines the lifetime of decorrelation events that can be quantified over long-path lengths in tissue (d∼1  cm).^11^ Encoding angular trajectory by the source is commonly done in microscopy and in fiber-optic delivery, where numerical aperture is tailored, but this is typically restricted to subdiffuse light sampling. It is possible that broader field angular source-encoding could have value with lens arrays or other delivery technologies. Spatial encoding of the source is commonly done in diffuse light sampling of tissue, such that the modeling can be simplified, and boundary matching or calibration of the model can be avoided through multi-distance methods.^12^ This is also an active area of development in optical methods such as Raman, fluorescence, and pulse oximetry.

### 3.2 Detector-Encoding Methods

The term “detector-encoding” here indicates that the imaging technique leverages a specifically designed detection scheme to isolate a desired optical signal. The range of detectors and detection techniques is almost as broad as sources, and many methods of diffuse light sampling use custom approaches to this, most of which are coupled to the source-encoding method as well. Temporal, wavelength, polarization, and coherence gating methods are all widely employed for various applications, and again wavelength is widely used for spectroscopic sampling of fluorescence, Raman, blood, or other chromophores. Several unique approaches to coherence gating exist, including Fourier transform methods in OCT^13^ and direct coherence gating methods in phase conjugation that extend the range of sampling to cm rather than mm.^14^ Spatial gating of the detection is perhaps the most widely deployed in confocal imaging where the simple definition of a pinhole or a light sheet can be used to define the volume sampled in the imaging. The extension of this to diffuse light has been illustrated to some degree by active illumination coupled to active detection, such as spatial frequency domain imaging (SFDI) methods, where the spatial Fourier distribution of light can be used to depth section tissue layers.^15^^,^^16^ Widefield coherence gating or polarization gating have not achieved the same level of success as SFDI at widefield sampling of tissue, although there could be innovations to be gained here. Wavelength encoding of detection can provide some sectioning capabilities as well, in examples, such as hyperspectral imaging^17^ or ultra-violet fluorescence methods.^18^

### 3.3 Population-Encoding Methods Within Tissue

The term “population-encoding” here indicates imaging methods that leverage the tagging of specific optical signals within the tissue of interest. These are perhaps the most scientifically compelling approaches that have captured the imagination of many researchers. Techniques such as acousto-optic tagging can be used to identify a tissue volume that is then interrogated by optical spectroscopy or transmission and thereby provide higher resolution of the volumes sampled. Even below the range of ultrasound, it is feasible to identify volumes within tissue by mechanical means, as in elastography OCT,^19^ or by vibrational excitation means, such as the Gruneisen coefficient^20^ or Brillouin spectroscopy.^21^ Coupling these tagging methods further to source- or detection-encoding of the features of subdiffuse or diffuse light may still provide unique opportunities for functional imaging of tissue. Related to this are methods where generation of light from within the medium can be used to tag regions, as has been shown by fluorescence, upconversion, or radioluminescence methods. In these methods, light is encoded by position of origin inside the medium. Again, there are likely ways to encode these unique spatial sources by other electromagnetic features of the light signal, such as polarization-encoding of fluorescence from within tissue, that yields information about the origins of the molecules producing it.^22^ Finding ways to encode space, time, angle, wavelength, polarization, or phase into uniquely tagged locations of light within tissue remains a scientific frontier to be developed.

**Fig. 4:**
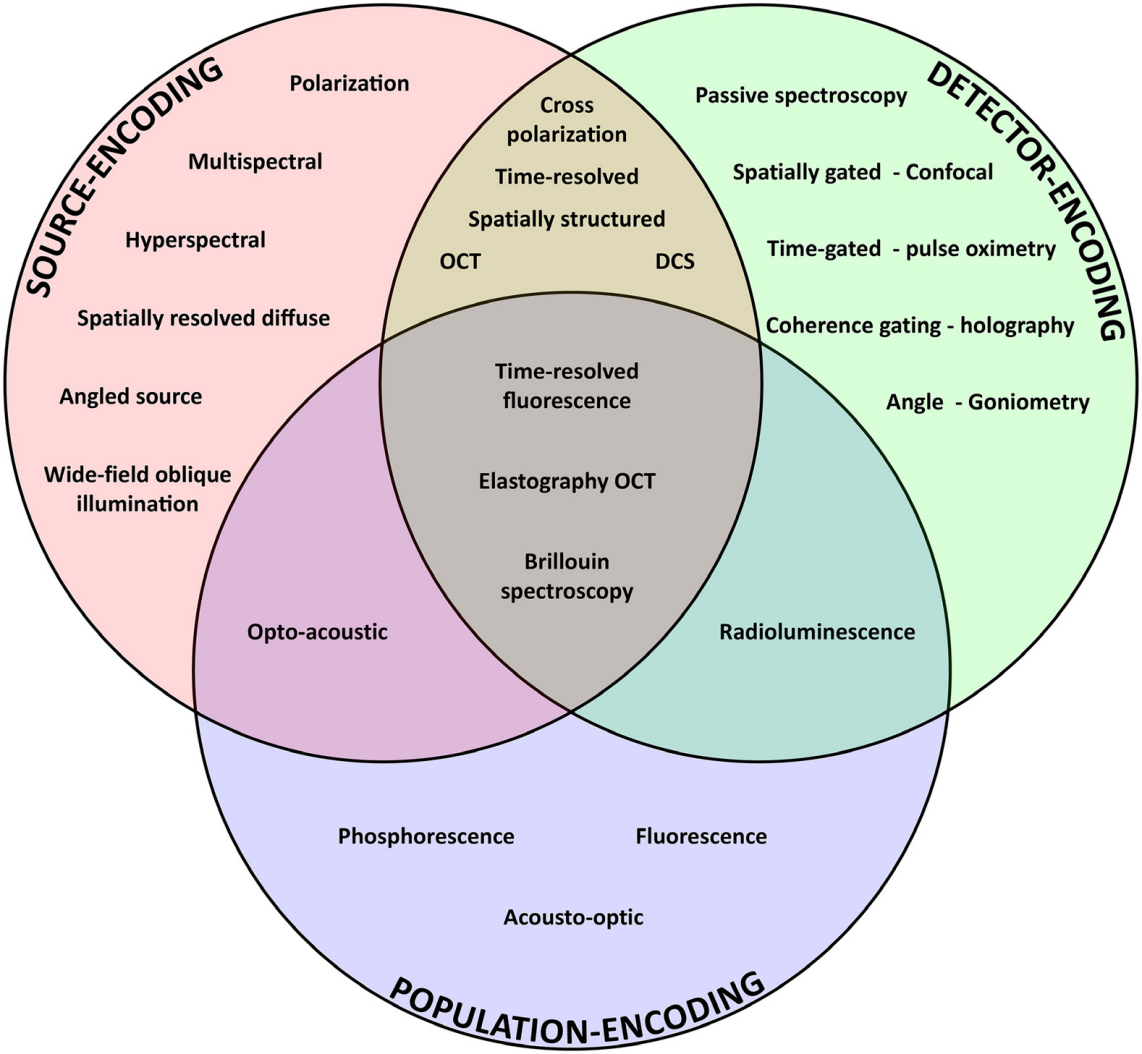
Venn diagram of a representative set of techniques of study in populations of photons, categorized by optical source-, detector-, and/or population-encoding scheme.

Figure 4 categorizes various optical measurement techniques by the source-, detector-, and/or population-encoding scheme employed. Only optical signal encodings are considered for methods that rely on non-optical excitation or detection (i.e., opto-acoustic, acousto-optic, radioluminescence). Many general techniques listed can be combined with other forms of encoding to selectively isolate subpopulations of photons. Importantly, all techniques in Fig. 4 employ one or more measurement parameters of light previously discussed (Figs. 1 and 2). Figure 4 also reinforces the point that source- and detector-encoding techniques are widely used today, but fewer established techniques leverage population-encoding to subsample diffusely scattered photons in tissue. This general framework for thinking about ways of encoding parameters of light into measurement techniques may inspire new ways of uniquely tagging optical signals within tissue.

## 4 Conclusion

Different subpopulations of photons in physical parameter space undergo diffusion as light is delivered into tissue. The mathematics of diffusion is useful for analyzing most of these parameters and generally follows Gaussian-distributed behavior. In some cases, a simple exponential expression effectively approximates the optical phenomenon of interest, and the decay parameters that govern the population changes can provide insight themselves on the light–tissue interaction. The predictable, diffusive nature of light transport in tissue provides ample opportunities for experimental and theoretical analysis, spanning space, time, angular trajectory, wavelength dispersion, electromagnetic polarization, and phase coherence, with a wide range of measurement or encoding techniques that subsample these distributions.
